# The Association of Suicidal Ideation With Firearm Purchasing During a Firearm Purchasing Surge

**DOI:** 10.1001/jamanetworkopen.2021.32111

**Published:** 2021-10-29

**Authors:** Michael D. Anestis, Shelby L. Bandel, Allison E. Bond

**Affiliations:** 1 New Jersey Gun Violence Research Center, Piscataway; 2 Rutgers, the State University of New Jersey, New Brunswick; 3 University of Southern Mississippi, Hattiesburg

## Abstract

**Importance:**

Firearm access is associated with risk for suicide, and, since early 2020, the US has experienced an unprecedented surge in firearm sales.

**Objective:**

To assess frequency of suicidal ideation among individuals who purchased firearms during the surge period (surge purchasers), other firearm owners, and non–firearm owners.

**Design, Setting, and Participants:**

Cross-sectional online survey data were collected from January to June 2021. Participants were recruited via quota sampling through Qualtrics Panels. Within Minnesota, zip codes from Minneapolis and St Paul were oversampled. Participants included 6404 US adults recruited from 3 states: New Jersey (n = 3197), Minnesota (n = 1789), and Mississippi (n = 1418). Participants identified as becoming a first-time firearm owner during the surge period, being an established firearm owner who purchased a firearm during the surge period, being a firearm owner who did not buy firearms during the surge period, and not owning firearms.

**Main Outcomes and Measures:**

The primary outcomes were lifetime, past year, and past month suicidal ideation as measured by the Self-injurious Thoughts and Behaviors Interview—Revised.

**Results:**

The mean (SD) age within the full sample was 44.81 (18.45) years, with 3132 males (48.8%), 4706 White adults (73.4%), 2674 reporting annual household income less than $50 000 (41.7%), and 1546 (24.1%) reporting current firearm ownership. In the full sample, individuals who purchased firearms during the surge period were more likely than were non–firearm owners to report lifetime suicidal ideation (odds ratio [OR], 2.21; 95% CI, 1.82-2.68), past-year suicidal ideation (OR, 2.10; 95% CI, 1.72-2.55), and past-month suicidal ideation (OR, 1.80; 95% CI, 1.41-2.29). In addition, among individuals who purchased firearms during the surge period, first-time owners were more likely than established firearm owners to report lifetime suicidal ideation (OR, 2.12; 95% CI, 1.43-3.14) and past-year suicidal ideation (OR, 2.37; 95% CI, 1.59-3.53). Results were largely consistent across states.

**Conclusions and Relevance:**

In this survey study, results highlighted that individuals who acquired firearms during the purchasing surge were more likely than other firearm owners and non–firearm owners to have experienced suicidal thoughts. This is particularly true for individuals who purchased a firearm for the first time during the surge period. This illustrates the need to implement policies and interventions that increase safety among firearm purchasers (eg, safe firearm storage) as well as those that promote the acquisition of alternative forms of protection (eg, home alarm systems).

## Key Points

**Question:**

Are individuals who purchased firearms during the 2020 to 2021 purchasing surge (surge purchasers) more likely than other firearm owners and non–firearm owners to have experienced suicidal ideation?

**Findings:**

Results of this survey study of 6404 adults sampled from New Jersey, Minnesota, and Mississippi indicated that surge purchasers were more likely than non–firearm owners to report lifetime, past year, and past month suicidal ideation. Among surge purchasers, first-time firearm owners were more likely than established firearm owners to report lifetime and past-year suicidal ideation.

**Meaning:**

These findings suggest that in contrast to other firearm owners, individuals driving the firearm purchasing surge from 2020 to 2021—particularly those who purchased a firearm for the first time—appear to be at elevated risk of having experienced suicidal thoughts.

## Teaser

This survey study assesses the frequency of suicidal ideation among individuals who purchased firearms during the 2020 to 2021 purchasing surge with other firearm owners and non–firearm owners.

## Introduction

Throughout 2020 and 2021, the US experienced multiple tumultuous events, including a pandemic, contentious presidential and state elections, and a racial justice movement that arose in response to police brutality toward Black, Indigenous, and other racial and ethnic minority communities. During and perhaps in response to these events, the US also experienced a surge in firearm sales.

Although it is difficult to determine national firearm sales figures accurately, various groups have provided estimates. For instance, based on background check data, an estimated 2 million firearms were purchased in March 2020,^1^ and more than 2.5 million Americans became first-time gun owners during the early months of 2020.^2^ The surge in firearm sales continued throughout 2020, with an estimated 23 million firearms sold,^3^ an increase from 2019 (13.9 million firearms sold) and 2018 (13.8 million firearms sold).^4^ This surge is not only notable because of its scale but also because firearm access is associated with gun violence–related outcomes, including suicide.^5,6,7^

Background check data provide a measurement of many legal firearm purchasing efforts; however, they do not necessarily equate to sales, thereby highlighting the need for data that more directly assesses purchasing behavior. Along these lines, a 2021 nationally representative study^8^ found that 6% of US residents purchased a firearm between March and mid-July 2020 (individuals purchasing firearms during the surge period), and of these, 34% were first-time purchasers, a rate higher than what is typical. Additionally, research to date examining individuals who purchased firearms for the first time during the surge period has found that nearly 40% of these individuals store at least 1 firearm unlocked,^9^ a concerning finding given the risk that accompanies unsafe storage (eg, suicide, unintentional shootings, theft).^10^

Notably, a prior study conducted by our team found that individuals purchasing firearms during the surge period had higher rates of lifetime, past-year, and past-month suicidal ideation relative to other firearm owners and non–firearm owners.^11^ To our knowledge, this is the only study to date examining this association, and the study did not differentiate between those who did and did not purchase firearms for the first time during the surge. The present study seeks to replicate and expand upon these findings. Specifically, the present study examined rates of lifetime, past-year, and past-month suicidal ideation among surge firearm purchasers, other firearm owners, and non–firearm owners. This work extended the findings of our previous survey primarily by comparing the frequency of suicidal thoughts between surge purchasers who acquired their first firearm and surge purchasers who acquired additional firearms. In doing so, the present study examines whether risk is not evenly distributed across all individuals purchasing firearms during the purchasing surge. The present study extends beyond previous work by examining a much larger sample comprising data from 3 specific states: Mississippi, Minnesota, and New Jersey. The states were chosen because they differ widely in geography, demographics, political climate, firearm ownership rates, firearm purchasing laws, gun violence rates, and culture.^12,13,14^ Our analyses thus allow for consideration of suicidal thoughts among various firearm owners who live in diverse environments, which are not concentrated in population centers often more heavily represented in national samples. As a secondary, exploratory aim, we will also examine firearm storage practices across firearm ownership groups to determine if surge purchasers overall and first-time firearm owners, in particular, are more prone to unsafe firearm storage. Should our findings demonstrate increased rates of suicidal ideation among surge purchasers more broadly and particularly among first-time firearm owners who purchased during the surge, this would highlight a trend of increasing environmental risk (ie, firearm access) among individuals at greater risk of developing suicidal thoughts.

## Methods

This survey study followed the American Association for Public Opinion Research (AAPOR) reporting guideline. All procedures were approved by the institutional review board at Rutgers University Health Sciences. Participants provided informed consent by selecting a box online indicating they understood the information in the consent document and agreed to participate, and they were compensated in the form and amount they agreed upon when they joined the panel (eg, airline miles, points at preferred retail outlets, cash, gift cards).

### Participants and Procedures

Participants included 6404 US residents recruited from 3 states—New Jersey (n = 3197), Minnesota (n = 1789), and Mississippi (n = 1418)—between January and June 2021 using Qualtrics Panels, an online survey platform that maintains a database of millions of US residents. Potential participants were informed that the primary aims of the study were to examine methods individuals use to keep themselves and their loved ones safe and to understand factors that influence the decision to obtain a firearm. Quota sampling was used to enroll a sample matched to 2010 state-level Census distributions for age, sex, race, ethnicity, income, and education. Recruitment in Minnesota included an oversampling of zip codes based within Minneapolis and St Paul (982; 54.6% of the statewide sample) in an effort to center this component of the project on the death of George Floyd. Although a precise participation rate is difficult to calculate, the estimate provided by Qualtrics was 54%. Target enrollment was 7500; however, Qualtrics reported delays in data acquisition during early 2021, and data collection was halted in June because of diminished returns (ie, 71.3% of the final sample had been obtained by late March, and 88.2% had been obtained by late April). Women were overrepresented in the full sample and in each statewide subsample; thus, sample weighting was applied. Weights were derived by dividing 2010 statewide Census demographic distributions by sample demographic distributions, such that the weight assigned to each participant was based on the state in which they lived. Weighted data are presented in all text and tables describing the sample. Demographic distributions can be found in Table 1.

**Table 1.** Demographic Descriptive Statistics for the Full Sample and Each Statewide Subsample^a^

Characteristic	No. (%)			
	Full sample, (N = 6401)	New Jersey, (n = 3197)	Minnesota, (n = 1789)	Mississippi, (n = 1418)
Age, mean (SD), y	44.81 (18.5)	45.63 (18.6)	46.39 (19.0)	40.95 (16.8)
Gender				
Male	3132 (48.8)	1567 (49.0)	894 (49.8)	670 (47.3)
Female	3217 (50.2)	1608 (50.3)	881 (49.0)	728 (51.3)
Transgender	33 (0.5)	11 (0.4)	8 (0.5)	13 (0.9)
Not male, female, or transgender	32 (0.5)	11 (0.4)	13 (0.7)	7 (0.5)
Sexual orientation				
Heterosexual	5473 (85.4)	2780 (86.9)	1521 (84.6)	1172 (82.7)
Gay or lesbian	186 (2.9)	96 (3.0)	48 (2.7)	42 (3.0)
Bisexual	378 (5.9)	160 (5.0)	125 (6.9)	93 (6.5)
Pansexual	59 (0.9)	22 (0.7)	24 (1.4)	12 (0.9)
Asexual	44 (0.7)	24 (0.7)	11 (0.6)	10 (0.7)
Other	66 (1.0)	26 (0.8)	18 (1.0)	23 (1.6)
Do not wish to disclose	201 (3.1)	87 (2.7)	51 (2.8)	64 (4.5)
Ethnicity				
Hispanic/Latinx	569 (8.9)	417 (13.1)	95 (5.3)	57 (4.0)
Racial identity				
American Indian/Alaskan Native	138 (2.2)	44 (1.4)	51 (2.9)	43 (3.0)
Asian	412 (6.4)	289 (9.0)	93 (5.2)	30 (2.1)
Black/African American	1086 (16.9)	459 (14.4)	133 (7.4)	494 (34.8)
Native Hawaiian/other Pacific Islander	40 (0.6)	18 (0.6)	15 (0.8)	7 (0.5)
White	4706 (73.4)	2325 (72.7)	1516 (84.3)	866 (61.0)
Other	220 (3.4)	151 (4.7)	49 (2.7)	21 (1.5)
Education				
Less than high school	191 (3.0)	71 (2.2)	57 (3.1)	63 (4.4)
High school diploma or equivalent	2469 (38.5)	1160 (36.3)	701 (39.0)	609 (42.9)
Associate’s degree	1097 (17.1)	445 (13.9)	376 (20.9)	277 (19.5)
Bachelor’s degree	1472 (23.0)	814 (25.5)	428 (23.8)	230 (16.2)
Master’s degree	949 (14.8)	582 (18.2)	182 (10.2)	184 (13.0)
Doctoral/professional degree	235 (3.7)	125 (3.9)	53 (3.0)	56 (3.9)
Annual household income, $				
<10 000	560 (8.7)	214 (6.7)	127 (7.0)	220 (15.5)
10 000-49 999	2114 (33.0)	840 (26.2)	618 (34.4)	657 (46.1)
50 000-99 999	1851 (28.8)	956 (29.9)	571 (31.7)	323 (22.9)
≥100 000	1887 (29.5)	1186 (37.1)	482 (26.8)	185 (15.4)
Current firearm owner	1546 (24.1)	495 (15.5)	522 (29.1)	528 (37.3)
Purchased firearm since March 2020	560 (8.7)	233 (7.3)	135 (7.5)	192 (13.5)
First-time firearm purchasers since March 2020	321 (57.5)	157 (67.9)	61 (45.3)	103 (53.6)
Suicidal ideation				
Lifetime	2257 (35.2)	1038 (32.5)	608 (33.8)	611 (43.1)
Past year	1549 (24.2)	703 (22.0)	420 (23.4)	425 (30.0)
Past month	738 (11.5)	315 (9.9)	192 (10.7)	231 (16.3)

^a^ Data presented in Table 1 represent weighted percentages.

Panel members were invited to participate in the survey via email, which included a link to the study landing page. Eligibility criteria included being age 18 years or older and residing in New Jersey, Minnesota, or Mississippi.

### Measures

#### Demographics

Demographic information was collected via items examining age, sex, race, annual household income, employment status, and status as an essential worker. Race and ethnicity were classified by participants and defined by the investigators. These variables were included to help assess the representativeness of the sample. Essential worker status was assessed by asking if they had been considered an essential worker at any point during COVID-19. We then asked participants to indicate what profession caused them to be labeled an essential worker. Essential worker status was included to control for the frequency with which individuals were required to spend time outside of their home and thereby potentially exposed to 1 or more of the events that characterized 2020 and the first half of 2021 (eg, COVID-19, the presidential election, racial justice protests, and increases in gun violence).

#### Firearms

Firearm ownership was assessed using a single item asking if they currently owned a firearm. Surge purchasing status was assessed using a single item that asked if they had purchased a firearm since March 2020. An additional question was asked to individuals who had purchased a firearm during the surge—was the firearm(s) purchased since March 2020 the first firearm(s) they had ever acquired. Firearm storage practices were assessed by asking firearm owners which of the following storage procedures they used for the firearms currently located in or around their homes. Answer choices included a gun safe, a locking device (eg, trigger lock or cable lock), hidden in closet or drawer unloaded, and hidden in closet or drawer loaded.

#### Suicidal Ideation

Suicidal ideation was assessed via the self-report version of the Self-injurious Thoughts and Behaviors Interview–Revised (SITBI-R).^15^ The SITBI-R assesses suicidal ideation by asking participants which, if any, of 8 different suicide-related thoughts they have experienced in their lifetime, the past year, and the past month. An individual was considered to have experienced suicidal ideation for a specific period if they endorsed any of the 8 suicide-related thoughts during that timeframe.

### Statistical Analysis

Between-group differences on lifetime, past-year, and past-month suicidal ideation rates were examined first in a series of χ^2^ analyses and then in a series of binary logistic regressions. In 1 series of regressions, surge firearm purchasers, other firearm owners, and non–firearm owners were compared for each of the 3 outcomes. In the second series of regressions, established firearm owners who purchased additional firearms during the surge were compared with first-time firearm owners who purchased during the surge on each of the 3 outcomes. Covariates included age, sex, Black racial identity, White racial identity, annual household income, past 12-month job loss, and status as an essential worker. Differences in storage practices were assessed using a series of χ^2^ analyses, with φ serving as an effect size index.

The statistical package SPSS version 26 (IBM) was used for data analysis. The logistic regressions used Wald χ^2^ tests to determine *P* values; χ^2^ analyses used the Pearson χ^2^ test to determine *P* values. All tests were 2-tailed, and statistical significance was set at *P* < .05. Analyses were performed from August to September 2020.

## Results

This study included 6401 adults with a mean (SD) age of 44.81 (18.5) years, 3132 males (48.8%), 3217 females (50.2%), 33 transgender individuals (0.5%), and 32 individuals identifying as neither male, female, nor transgender (0.5%). The sample included 1086 (16.9%) Black or African American individuals and 4706 (73.4%) White individuals (Table 1).

### Comparison of Surge Purchasers, Other Firearm Owners, and Non–Firearm Owners

Results from univariate analyses indicated individuals who purchased firearms during the surge period (341 [56.1%]) were more likely than non–firearm owners (1629 [34.3%]), who were more likely than other firearm owners (313 [28.6%]), to have experienced lifetime suicidal thoughts (χ^2^ = 129.47, *P* < .001, φ = 0.14). Similarly, surge purchasers (235 [42.0%]) were more likely than non–firearm owners (1118 [23.5%]) to report past-year suicidal ideation, and non–firearm owners were more likely than other firearm owners (196 [17.9%]) to report past-year suicidal ideation (χ^2^ = 121.12, *P* < .001, φ = 0.14). Lastly, surge purchasers (115 [20.5%]) were more likely than non–firearm owners (548 [11.5%]) to report past-month suicidal ideation, and non–firearm owners were more likely than other firearm owners (75 [6.9%]) to report past-month suicidal ideation (χ^2^ = 67.96, *P* < .001, φ = 0.10).

Multivariate analyses yielded similar results. Surge purchasers were more likely than non–firearm owners to report lifetime suicidal ideation (OR, 2.21; 95% CI, 1.82-2.68), past-year suicidal ideation (OR, 2.10; 95% CI, 1.72-2.55), and past-month suicidal ideation (OR, 1.80; 95% CI, 1.41-2.29). Other firearm owners did not differ from non–firearm owners about lifetime or past-year suicidal ideation but were less likely than non–firearm owners to report past-month suicidal ideation (OR, 0.76; 95% CI, 0.58-0.99) (Table 2).

**Table 2.** Logistic Regressions Examining the Association Between Firearm Ownership Group (Surge Purchaser, Non–Surge Firearm Owners, Non–Firearm Owners) and Suicidal Ideation (Lifetime, Past Year, Past Month) Within the Full Sample

Characteristic	b (SE)	OR (95% CI)
**Lifetime ideation**		
Age	−0.04 (0.00)	0.96 (0.96 to 0.97)
Black	−0.12 0(.01)	0.89 (0.73 to 1.08)
White	0.23 (0.09)	1.26 (1.06 to 1.50)
Sex	0.16 (0.06)	1.17 (1.06 to 1.31)
Annual household income	−0.06 (0.01)	0.94 (0.93 to 0.96)
Past 12-mo job loss	0.45 (0.07)	1.57 (1.36 to 1.82)
Essential worker	0.23 (0.07)	1.26 (1.12 to 1.41)
Firearm purchaser status		
Non–surge firearm owners	0.03 (0.08)	1.03 (.88 to 1.21)
Surge firearm purchasers	0.79 (0.10)	2.21 (1.82 to 2.68)
**Past-year ideation**		
Age	−0.04 (0.00)	0.96 (0.96 to 0.96)
Black	−0.08 (0.11)	0.92 (0.75 to 1.14)
White	0.30 (0.10)	1.35 (1.12 to 1.62)
Sex	0.04 (0.07)	1.04 (0.92 to 1.18)
Annual household income	−0.05 (0.01)	0.95 (0.93 to 0.97)
Past 12-mo job loss	0.41 (0.08)	1.50 (1.29 to 1.75)
Essential worker	0.16 (0.07)	1.18 (1.04 to 1.34)
Firearm purchaser status		
Non–surge firearm owners	−0.08 (0.09)	0.93 (0.77 to 1.11)
Surge firearm purchasers	0.74 (0.10)	2.10 (1.72 to 2.55)
**Past-month ideation**		
Age	−0.04 (0)	0.96 (0.95 to 0.96)
Black	−0.08 (0.13)	.93 (0.71 to 1.20)
White	0.19 (0.12)	1.21 (0.96 to 1.53)
Sex	0.05 (0.09)	1.05 (0.89 to 1.24)
Annual household income	−0.07 (0.01)	0.93 (0.91 to 0.95)
Past 12-mo job loss	0.38 (0.09)	1.47 (1.22 to 1.77)
Essential worker	0.27 (0.08)	1.31 (1.11 to 1.55)
Firearm purchaser status		
Non–surge firearm owners	−0.28 (0.14)	0.76 (0.58 to 0.99)
Surge firearm purchasers	0.59 (0.12)	1.80 (1.41 to 2.29)

Abbreviation: OR, odds ratio.

### Comparison of First-Time Firearm Owners and Established Firearm Owners Who Purchased Firearms During the Purchasing Surge

In our univariate analyses examining only surge purchasers, first-time firearm owners were more likely than established firearm owners to report lifetime suicidal ideation (213 [66.6%] vs 99 [41.8%]; χ^2^ = 33.96; *P* < .001; φ = 0.25), past-year (170 [53.1%] vs 64 [27.0%]; χ^2^ = 38.13; *P* < .001; φ = 0.26) and past month suicidal ideation (78 [24.3%] vs 37 [15.6%]; χ^2^ = 6.29; *P* = .01; φ = 0.11).

Multivariate analyses yielded similar results. First-time firearm owners were more likely than established firearm owners to report lifetime suicidal ideation (OR, 2.12; 95% CI, 1.43-3.14) and past-year suicidal ideation (OR, 2.37; 95% CI, 1.59-3.53). The 2 groups did not differ on past-month suicidal ideation (OR, 1.38; 95% CI, 0.86-2.21) (Table 3).

**Table 3.** Logistic Regressions Examining the Association Between Surge Purchaser Group (First-Time Firearm Owner, Established Firearm Owner) and Suicidal Ideation (Lifetime, Past Year, Past Month) Within the Full Sample^a^

Characteristic	b (SE)	OR (95% CI)
**Lifetime ideation**		
Age	−0.03 (0.01)	0.97 (0.95 to 0.98)
Black	−0.21 (0.37)	0.81 (0.40 to 1.66)
White	−0.63 (0.33)	0.54 (0.28 to 1.01)
Sex	−0.37 (0.22)	0.69 (0.45 to 1.06)
Annual household income	0.02 (0.03)	1.02 (0.96 to 1.09)
Past 12-mo job loss	1.87 (0.28)	6.47 (3.71 to 11.30)
Essential worker	0.07 (0.21)	1.07 (0.71 to 1.62)
First-time firearm owner vs established firearm owner	0.75 (0.20)	2.12 (1.43 to 3.14)
**Past year ideation**		
Age	−0.04 (0.01)	0.97 (0.95 to 0.98)
Black	0.35 (0.34)	1.41 (0.73 to 2.74)
White	−0.14 (0.31)	0.87 (0.48 to 1.58)
Sex	−0.49 (0.22)	0.61 (0.40 to 0.93)
Annual household income	0.04 (0.03)	1.04 (0.98 to 1.11)
Past 12-mo job loss	1.25 (0.23)	3.49 (2.21 to 5.50)
Essential worker	0.08 (0.21)	1.08 (.71 to 1.64)
First-time firearm owner vs established firearm owner	0.86 (0.20)	2.37 (1.59 to 3.53)
**Past month ideation**		
Age	−0.03 (0.01)	0.98 (0.96 to 1.00)
Black	0.06 (0.36)	1.06 (0.52 to 2.17)
White	−0.29 (0.34)	0.75 (0.39 to 1.45)
Sex	−0.04 (0.24)	0.96 (0.60 to 1.56)
Annual household income	0.03 (0.04)	1.03 (0.96 to 1.11)
Past 12-mo job loss	0.90 (0.25)	2.46 (1.52 to 3.97)
Essential worker	0.08 (0.25)	1.08 (0.66 to 1.76)
First-time firearm owner vs established firearm owner	0.32 (0.24)	1.38 (0.86 to 2.21)

Abbreviation: OR, odds ratio.

^a^ Given differences between the 3 states in terms of rurality, zip code data was recoded to calculate population density, which was then used to categorize rural or urban status. In univariate analyses, rural or urban status was associated with all 3 suicidal ideation outcomes as well as with the firearm groups. Primary analyses were rerun including this variable as a covariate. In these multivariate analyses, the results were unchanged, and rural or urban status was not associated with any outcome. Thus, the variable in the primary analyses was not included.

### Firearm Storage Analyses

Surge purchasers were more likely than other firearm owners to endorse the use of locking devices (204 [36.4%] vs 289 [26.4%]; χ^2^ = 17.65; *P* < .001; φ = 0.10) and less likely to endorse storing unloaded firearms hidden in a closet or drawer (127 [22.7%] vs 322 [29.5%]; χ^2^ = 8.61; *P* = .003; φ = −0.07). Surge purchasers and other firearms owners did not differ in the frequency of endorsing the use of gun safes (250 [44.6%] vs 466 [42.6%]; χ^2^ = 0.61; *P* = .44; φ = 0.02) or keeping loaded firearms hidden in a closet or drawer (77 [13.8%] vs 175 [16.0%]; χ^2^ = 1.43; *P* = .23; φ = −0.03).

Among surge purchasers, first-time firearm owners were less likely than established firearm owners to endorse the use of gun safes (125 [38.9%] vs 125 [52.7%]; χ^2^ = 10.50; *P* = .001; φ = −0.14) or storing loaded firearms hidden in a closet or drawer (35 [10.9%] vs 43 [18.1%]; χ^2^ = 5.94; *P* = .02; φ = −0.10). In contrast, first-time firearm owners were more likely to endorse the use of locking devices to store firearms (135 [42.1%] vs 69 [29.1%]; χ^2^ = 9.85; *P* = .002; φ = 0.13). The 2 groups did not differ in terms of storing unloaded firearms hidden in a closet or drawer (73 [22.7%] vs 54 [22.8%]; χ^2^ = 0.00; *P* = .99; φ = 0.00).

## Discussion

The primary aim of this study was to examine rates of suicidal ideation among first-time and established surge firearm purchasers, other firearm owners, and non–firearm owners. Additionally, we aimed to examine this within 3 states that vary widely in terms of their residents’ demographic and cultural backgrounds, geographic location, the strictness of their firearm legislation, and their rates of firearm ownership and gun violence. Our results were largely consistent with our hypotheses and thus highlight that individuals who purchased firearms since March 2020—in contrast to other firearm owners and non–firearm owners—were associated with experiencing suicidal thoughts at an elevated frequency.

These findings echo those from our prior study,^11^ but expand upon them in several ways. First, we differentiated between established firearm owners who purchased additional firearms during the purchasing surge and those who became firearm owners for the first time during that timeframe. Across all analyses, first-time owners were more likely than established owners to report lifetime and past-year suicidal thoughts in both univariate (φ, 0.21-0.35) and multivariate (OR, 2.12-4.12) analyses. However, this difference only held for past-month suicidal thoughts in the univariate analyses.

These findings suggest that individuals who decided to become firearm owners during the 2020 to 2021 purchasing surge exhibit a higher risk for suicidal thoughts than typical firearm owners. As such, the introduction of environmental risk (ie, the presence of a firearm) becomes substantially more dangerous because the odds of within-individual risk (active suicidal thoughts) coinciding with that environmental risk become far greater over time. The fact that suicidal thoughts were particularly common among surge purchasers who became first-time firearm owners is an important consideration given data demonstrating an elevated suicide rate in the months following the first acquisition of a firearm.^16^ Thus, these findings speak to several vital points for intervention. First, a heavy emphasis on safe firearm storage and temporary firearm storage away from home during times of stress must be articulated to the customer at the point of sale to prompt behavior that mitigates environmental risk. This approach, which emphasizes harm reduction, needs to be supplemented with practical tools, such as information about safe firearm storage options, incentives for both retailer and consumer to purchase safe storage equipment, and information about where firearm owners can legally and temporarily store firearms outside the home.^17,18^ In terms of policy, lawmakers may want to consider safe storage bills. A study by the RAND Corporation reported that implementation of child access laws, a specific form of safe storage legislation, exhibited a particularly strong association with subsequent decreases in firearm deaths.^19^ Additional policy-based changes worth considering in light of these findings include purchasing delays (eg, waiting periods) and mandating suicide risk screening questions during firearm purchases. Furthermore, these findings highlight the potential value in incentivizing the acquisition of alternative modes of home protection (eg, home alarm systems). This approach, which emphasizes upstream prevention, would acknowledge the motivation for feeling safe at home while offering options that include lower risk profiles.

In our exploratory analyses, we examined storage practices across firearm ownership groups. Surge purchasers were more likely than other firearm owners to use locking devices (eg, cable locks, trigger locks) and less likely to store unloaded firearms hidden in a closet or drawer. Among surge purchasers, first-time firearm owners were more likely than established firearm owners to use locking devices and less likely to store loaded firearms hidden in a closet or drawer or to use gun safes. These results cannot provide a comprehensive picture of storage habits because we could not assess the complete storage tendencies for each firearm owned by each participant. Nonetheless, the picture that emerged from these data does not indicate that first-time or established firearm owners who purchased during the surge period were associated with storing their firearms less safely than other firearm owners. Considered within the context of the suicidal ideation findings, our findings suggest that those most vulnerable to suicidal thoughts are not maintaining the most ready access to their firearms. However, it is worth noting that all groups of firearm owners endorsed fairly low rates of safe firearm storage, thereby highlighting the importance of increasing the implementation of safe storage practices across firearm ownership communities.

### Limitations

This study had limitations. First, using quota sampling rather than probability-based sampling limits generalizability and precludes us from providing estimates of firearm purchasing rates in each state. Second, these findings are based on self-report and have not been validated. Third, we were unable to determine whether the firearms purchasers during the surge represented the first firearms in the home or merely represented the first firearm that the respondents considered to be their own. This information has implications for how to design interventions and prevention efforts.

## Conclusions

In this study, individuals who purchased a firearm during the 2020 to 2021 purchasing surge were more likely than other firearms owners and non–firearm owner to have experienced suicidal thoughts. We believe the results of this survey study provide important information that highlights the need to develop and implement strategies that can mitigate suicide risk among surge firearm purchasers, particularly among those purchasing a firearm for the first time. The increased rate of suicidal ideation among surge purchasers does not mean the nation is destined to have a surge in firearm suicides, but it indicates an increased risk in a manner that requires our careful attention.
